# Non-coding RNA modulation in osteoclasts and its implications for osteoblast lineage cell behavior in a co-culture system

**DOI:** 10.1186/s12964-025-02324-7

**Published:** 2025-08-12

**Authors:** Sara Reis Moura, Jacob Bastholm Olesen, Martin Lindberg-Larsen, Mário Adolfo Barbosa, Kent Søe, Maria Inês Almeida

**Affiliations:** 1 i3S - Instituto de Investigação e Inovação em Saúde, Universidade do Porto, Porto, Portugal; 2 INEB - Instituto de Engenharia Biomédica, Universidade do Porto, Porto, Portugal; 3 Department of Pathology, Odense University Hospital, Odense, Denmark; 4 Clinical Cell Biology, Pathology Research Unit, Department of Clinical Research, University of Southern Denmark, Odense, Denmark; 5 Department of Orthopaedic Surgery and Traumatology, Odense University Hospital, Odense, Denmark; 6 Orthopaedic Research Unit, Department of Clinical Research, University of Southern Denmark, Odense, Denmark

**Keywords:** Bone remodelling, LncRNA, MiRNA, Cytokines/chemokines, Extracellular matrix, Molecular engineering

## Abstract

The interplay between osteoblasts and osteoclasts (OCs) is a highly regulated and coordinated process essential for maintaining bone skeletal integrity and health. Disruption of this balance marks the onset of various bone disorders, such as osteoporosis. In our previous study, we demonstrated that non-coding RNAs (ncRNAs) were able to regulate OC behaviour. Specifically, the long ncRNA *DLEU1* and the small ncRNA miR-16 independently control OCs multinuclearity, fusion and resorption. Herein, we investigate whether OCs modulated with ncRNAs impact the behaviour of osteoblast lineage cells (OBs) in a biomimetic co-culture system. Communication between OB and OC comprises numerous molecules and processes, including the secretion of coupling factors, release of matrix-derived signals during bone resorption, and exposure of previously occluded proteins within the bone resorption cavities. Firstly, we explored how ncRNAs influence the composition of the secretome and the remodelling of resorption cavities left by the ncRNA-engineered-OCs on OBs responses. In our OC/OB co-culture system, conducted in the absence of FBS and exogenous RANKL, we observed changes in the levels of secreted factors 72 h after *DLEU1* levels were decreased in mature OCs (siDLEU1-OC condition), specifically in RANKL, leptin, osteocalcin, MMP7, MMP8, TIMP1 and TIMP2. Meanwhile, leptin and MMP8 levels were increased in the OC/OB co-culture when miR-16 was overexpressed in OCs (miR-16-OC condition). The results also show that OBs co-cultured with siDLEU1-OCs exhibited impaired migratory capacity and increased ALP levels, while OBs co-cultured with miR-16-OCs displayed a tendency towards decreased elongation. On bone slices, OBs persisted for a longer duration in the trenches created by siDLEU1-OCs. Besides altering OBs behaviour, silencing of *DLEU1* reduced the area of each individual trench cavity, while miR-16 overexpression significantly hindered the total eroded surface and inhibited trench formation in the OC/OB co-culture system. Overall, these findings identify *DLEU1* and miR-16 as key regulators in the interplay between human primary OBs and OCs, highlighting the potential of targeting ncRNAs in OCs to modulate their paracrine and matrix-mediated effects on OBs.

**Supplementary Information:**

The online version contains supplementary material available at 10.1186/s12964-025-02324-7.

## Introduction

In the dynamic bone microenvironment, there is a delicate equilibrium between osteoclast (OC)-mediated bone resorption and osteoblast-driven bone formation, which orchestrates the continuous process of bone remodelling. These complementary processes are pivotal for maintaining the size, structure, and mineral content of the bone [1]. This process encompasses three successive phases: (1) an initial resorption phase performed by the OCs; (2) a reversal phase, during which osteoblasts are recruited and alternate activity with OCs; and (3) a final bone formation phase [2–7]. These stages may partially overlap both temporally and spatially [8]. Disruptions in any phase of bone remodelling, caused by several intrinsic or extrinsic factors can adversely affect subsequent phases, influencing the onset of various bone-related diseases [9]. These factors range from hormone deficiency, such as oestrogen, parathyroid hormone (PTH) and vitamin D_3_ [10–12], to aging, lifestyle choices (including sedentarism, smoking, alcohol consumption), and dietary deficiencies [13], all of which can contribute to OC activation. This is evident in osteoporosis, which is characterized as a condition where OC bone resorptive activity exceeds the bone formation capacity [9, 14, 15], resulting in increased bone fragility and risk of fractures after low energy trauma [15]. The duration of the resorption/reversal phase depends on the recruitment of osteoprogenitor cells, and bone formation is only initiated once a critical cell density is reached, highlighting the delicate balance in this process [6]. Accordingly, a diminished rate of osteoprogenitor recruitment leads to an extended duration of the reversal phase, thereby exacerbating bone degradation [6]. Therefore, studying the molecular drivers of this dynamic mechanism is important, as it may determine whether bone resorption, at a particular site, should continue, remain unchanged, or be replaced by new bone formation.

We recently demonstrated that OC multinucleation and bone resorption is modulated by non-coding RNAs (ncRNAs). Specifically, we showed that the long ncRNA *DLEU1* (Deleted In Lymphocytic Leukemia 1) and the small ncRNA miR-16, located in the chr 13q14 region [16, 17], have an impact on the fusion and resorptive capacity of OCs through distinct mechanisms, targeting different stages of differentiation and maturity. Specifically, both *DLEU1* inhibition and miR-16 overexpression hinder the OCs’ fusion [18]. Since multinucleation is often positively correlated with OCs aggressiveness [19–21], this reduction may suggest an impairment of the OCs activity. In fact, modulation of the levels of these ncRNAs in mature OCs affects their resorption mode, characterized by distinct features and holding clinical relevance [22–24]. Herein, we hypothesize that reducing *DLEU1* and increasing miR-16 levels in mature OCs would influence the behaviour of OBs. To test this hypothesis, a co-culture system using human primary OCs and OBs on bone slices was employed to better mimic the physiological bone microenvironment.

The coupling between OCs and osteoblasts, with opposing but complementary functions, involves a bidirectional communication essential for maintaining bone homeostasis. This communication occurs through paracrine effects involving e.g., the secretion of coupling factors, such as cytokines [25, 26], growth factors [26] and ncRNAs [27, 28], either free or entrapped within extracellular vesicles, along with matrix-derived signals released during bone resorption, or through direct cell-cell interactions [29–31]. Nevertheless, a deeper understanding of the regulatory networks governing skeletal homeostasis remains necessary.

Despite therapeutic advances, current strategies for bone diseases remain suboptimal as antiresorptive agents (e.g., bisphosphonates and denosumab) can cause unintended remodelling arrest, potentially leading to atypical fractures and impaired bone repair, while anabolic therapies (e.g., teriparatide and romosozumab) stimulate bone formation but are limited by short-term efficacy, high cost, and cardiovascular safety concerns [32–35]. Moreover, most of these drugs act unidirectionally, either inhibiting resorption or promoting bone formation without restoring the natural coupling between OCs and osteoblasts. This imbalance can lead to compensatory effects, such as rebound bone loss after discontinuation of therapy or increased fracture risk in some patients [35, 36]. Given these challenges, there is a growing interest in approaches that can simultaneously modulate both bone resorption and formation. In this context, ncRNAs offer a promising avenue, as they act at multiple regulatory levels and can potentially modulate both osteoblast and OC functions simultaneously, offering a dual-action mechanism that could re-establish physiological coupling and provide a more nuanced and adaptive strategy for bone disease management [27]. Considering this, our study aims to explore the impact of siDLEU1- and miR-16-engineered OCs on osteoblast lineage cells (OBs) behaviour and activity, providing insights into how ncRNA-based strategies could offer a promising approach to treat bone diseases.

## Materials and methods

### Generation of human OCs and modulation of ncRNA levels

Human primary monocytes were isolated from buffy coats (BC) obtained from anonymous human blood donors (50–65 years old males), who provided informed consent at the Odense University Hospital (Denmark), using immunomagnetic separation through BD IMag Anti-Human CD14 Magnetic Particles – DM (BD Biosciences, San Jose, USA), and differentiated into OCs, as previously described [18, 19, 37, 38]. Briefly, newly isolated monocytes were seeded at a cell density of 6.7 × 10^4^ cells/cm^2^ in α-MEM (Invitrogen, Taastrup, Denmark) supplemented with 25 ng/mL M-CSF (R&D Systems, Abingdon, UK) for 2 days. Following the initial 2-day culture, cells were differentiated into mature OCs over 6 additional days in fresh medium containing 25 ng/mL M-CSF and 25 ng/mL RANKL (R&D Systems, Abingdon, UK).

Matured OCs (8 days post-monocyte isolation) were transfected using the GenMute™ siRNA transfection reagent for primary macrophages (SL100568-PMG, SignaGen Laboratories, Frederick, USA), as previously described by us [18]. Transfection complexes were prepared by incubating the reagent for 15 min with either a silencing RNA against Deleted In Lymphocytic Leukemia 1 (DLEU1) (siDLEU1; 25 nM; Lincode Human DLEU1 siRNA – SMARTpool; R-0200009-00-0005; Dharmacon - Horizon Discovery, Lafayette, USA), miR-16 mimic (miR-16; 50 nM; mirVana^®^ miRNA mimic; MC10339; ThermoFisher, Waltham, USA) or the respective negative controls [Lincode Non-targeting Pool (CTR; 25 nM; D-001320-10-05; Dharmacon - Horizon Discovery) and mirVana™ miRNA Mimic, Negative Control #1 (SCR; 50 nM; 4464058; ThermoFisher)]. These transfected OCs are referred as tOCs. siGLO Red Transfection Indicator (25 nM; Dharmacon – Horizon Discovery) was used to confirm successful transfection and to measure the transfection efficiency (Supplementary Material and Methods; Supplementary Results and Supplementary Figure S1).

### Generation of osteoblast lineage cells (OBs) and culture in vitro

Human OBs were sourced from the removed femoral neck and head bone tissue obtained from a single donor undergoing hip replacement surgery at the Odense University Hospital, after being granted ethical approval by the local ethics committee (S-20110114) [31, 39]. Exclusion criteria were clear signs of osteoporosis and treatment with glucocorticoids within the previous month prior to surgery. All the procedures are in agreement with the Declaration of Helsinki and were performed as previously described [31, 40]. Briefly, bone specimens were initially sectioned into smaller fragments and thoroughly cleansed with PBS 1x through two cycles of vigorous shaking. Subsequently, five to six bone slices were meticulously positioned within 12-well plates. A metal grid was placed on top of the bone slices to prevent any movement. The culture medium used was DMEM (Sigma-Aldrich, St Louis, USA), enriched with 10% (v/v) FBS (Sigma-Aldrich), 2 nM L-glutamine (Sigma-Aldrich), 50 µg/mL ascorbic acid (catalog number: A0278; Sigma-Aldrich), 10 mM β-glycerophosphate (catalog number:50020; Sigma-Aldrich), and 10^− 8^ M dexamethasone (catalog number: D4902; Sigma-Aldrich). Cells were cultured for 7 days in a controlled environment, at 37 °C with 5% (v/v) CO_2,_ and the media was renewed every 7 days, allowing the outgrowth cells to migrate from the surface of the bone fragments to the culture plate. Following 14 days of culture, the metal grid and the bone fragments were removed and the media renewed twice a week. Cells obtained through this method maintained their proliferative capacity under osteogenic-inducing conditions (β-glycerophosphate, ascorbic acid and dexamethasone) and were used until passage 8. Moreover, they express both mRNA [31] and protein [41] levels of collagen type I, produce RANKL [41] and show ALP activity [41] (established OB lineage markers [42]).

### Bone resorption assays in co-cultures of transfected mature OCs with OBs 

tOCs were detached using accutase (Biowest, Nuaillé, France) and reseeded on top of 0.4 mm thick bovine cortical bone slices (Boneslices.com, Jelling, Denmark; placed individually in a 96-well plate), at a density of 5 × 10^4^ cells/bone slice (5 bone slices/condition). After 4 h in culture with α-MEM supplemented with 25 ng/mL M-CSF, 12 500 OBs were seeded on top of the OCs and the bone slice, and cultured for 3 additional days [31]. The secretome of these co-cultures was collected and stored at -80 °C for subsequent analysis of changes in its composition. Resorption events were visualized and quantified following toluidine blue staining, as previously described [43]. Briefly, bone slices were rinsed with distilled water, the adherent cells were scraped from the bone surface (using a cotton swab), and rinsed once again with distilled water. The bone slices were then blotted dry on paper and stained for 15 s in a 0.01 g/mL filtered toluidine blue solution (pH ~ 7.0; diluted in a 0.01 g/mL sodium borate solution). Bone slices were blotted on filter paper to remove excess stain before imaging and analysis. Subsequently, the samples were examined using light microscopy to determine the percentage of eroded surface and classify the resorption cavities (whether pits or trenches, as defined in the literature [44, 45]). The percentage of bone surface area covered by resorption cavities was calculated by dividing the number of grid squares containing stained cavities by the total number of squares examined. These quantifications were conducted with a 100-point grid graticule (catalog number:01A24.5075; Graticules Optics, Tonbridge, UK) [45, 46]. Quantitative analyses were performed in a blinded manner concerning the treatment conditions.

### ALP measurement

After 3 days of co-culturing OB with tOCs onto bone slices, ALP activity was measured using a colorimetric assay with 4-nitrophenyl phosphate (4-NNP; Fluka Chemie; Darmstadt, Germany) as substrate, as previously described [40]. Briefly, after being washed, cells were incubated with a reaction buffer, containing 0.06 M Na_2_CO_3_ (Acros Organics, Geel, Belgium), 0.04 M NaHCO_3_ (Acros Organics), 0.1% (v/v) TritonX-100 (Merck KGaA, Darmstadt, Germany), 2 mM MgSO_4_ (Sigma-Aldrich) and 6 mM 4-NNP, in the dark for 30 min and 37 °C. Then, a stop solution of 1 M NaOH (VWR chemicals, Leuven, Belgium) was added in a 1:1 ratio and the absorbance read for the wavelengths of 405–645 nm using the spectrophotometer microplate reader Synergy MX (Biotek Synergy, Winooski, USA).

### Protein quantification in the secretome

Secretome of tOC/OB co-cultures was collected after 3 days. The levels of 26 bone-related proteins were measured using the Human Bone 13-Plex Discovery Assay^®^ Array (HDBN13) and Human MMP and TIMP Discovery Assay^®^ Array for Cell Culture and non-blood samples (HMMP/TIMP-C, O), at Eve Technologies Corp (Calgary, Canada). The levels of RANKL in the secretome were determined using the commercial assays Human TRANCE/RANK L/TNFSF11 DuoSet ELISA (Y626; R&D Systems) and DuoSet ELISA Ancillary Reagent Kit 2 (DY008B; R&D Systems), according to the manufacturer’s instructions. Absorbance was measured at 450 nm. Concentrations were calculated based on standard curves. Results are presented as fold-change relative to the concentration measured in the control condition (displayed as bar plots, with individual donor values represented by dots), as well as absolute concentrations shown in heatmaps.

### Time-lapse recordings of co-cultures of transfected mature OCs with OBs

Time-lapse recordings were performed in the tOCs co-cultured with untransfected OBs seeded on 0.2-mm-thick bone slices, as aforementioned and described elsewhere [44, 47]. The bone slices were stained for 1 h with 0.25 mg/mL N-hydroxysuccinimide ester-activated rhodamine fluorescent dye (diluted in a 0.1 M bicarbonate buffer; ThermoFisher) and washed once with α-MEM before cell seeding. tOCs, previously detached using accutase, were resuspended in α-MEM serum-free media containing 100 nM SiR-actin and 10 µM verapamil (Spirochrome, Stein am Rhein, Switzerland) supplemented with M-CSF, and reseeded (5 × 10^4^ cells/bone slice) onto rhodamine-stained bone slices (each placed beforehand individually in 96-well plate). After 4 h, the OBs labelled for 20 min with 5 μm Vybrant DiO (Invitrogen, Taastrup, Denmark), were seeded (12 500 cells) onto the bone slice (with the tOC already attached) and left to adhere for 2 h. The bone slices were subsequently transferred into an eight-wells chambered cover-glass (Nunc Lab-Tek II; ThermoFisher) with α-MEM supplemented with 25 ng/mL M-CSF, 10 nM SiR-actin and 10 µM verapamil, and placed in the incubation chamber of the confocal Olympus Fluoview FV10i microscope (Olympus Corporation, Tokyo, Japan) with 5% (v/v) CO_2_ at 37 °C, for 3 uninterrupted days. Cells were visualized through a 10x objective lens with a confocal aperture set to 2.0, corresponding to a z-plane depth of 20.2 μm [41] and covering surface areas 0.3 to 0.4 mm^2^ (size of each field recorded) [47]. To ensure comprehensive data collection, three different random fields were recorded over a continuous period of three days for each condition and donor.

For the assessment of the resorbed area and study of the impact of co-culturing tOCs with OBs, and on the interaction between OCs and OBs, the FV10-ASW 4.1/4.2 Viewer software by Olympus and ImageJ were used [48]. Measurement of the OBs’ migration was performed over the course of 72 h using the *Manual Tracking* plugin from ImageJ. OBs that did not remain within the recording window for the duration of the 72 h were excluded from the analysis. The size and morphology parameters of the OBs were acquired by manually outlining all the OBs present in the recording window after 72 h of co-culture with tOCs, using the *Measure* tool from ImageJ. Each OB was tracked over the course of the recordings to count the number of cell division events. The categorization of the resorption events followed previously described criteria [18, 47, 49]. The extent of resorption for each event was quantified by delineating the perimeters of the resorption cavities (black regions on the Rhodamine staining) using ImageJ software. To gauge the impact of the newly exposed matrix (resorbed cavities) on OBs behaviour, the time an OB interacted with the resorbed cavities (> 50% cell surface over/inside the excavation), as well as the number of OBs within the cavities (> 50% cell surface over/inside the excavation), was analysed over the course of 72 h. Only OBs that remained within the cavities for more than 3 frames (one frame ~ 31.4 min) were considered.

Data was gathered from four distinct OC donors, each with four different experimental conditions (siDLEU1, miR-16 mimics or their corresponding controls) and co-cultured with non-transfected OBs. In total, analyses of 216 h time-lapse recordings were performed for each condition and donor, amounting to 864 h when considering all four experimental conditions (per donor).

### Statistical analysis

All graphs were generated using GraphPad Prism software (version 9, GraphPad Software) and are represented as dot plots. For *N* ≤ 5 independent experiments, non-parametric distribution was assumed. For *N* > 5, data sets were tested for normality using the Shapiro-Wilk tests. For data that passed the normality test, comparisons between two groups were made using the Student’s *t* test, with results presented as means. For data displaying a non-normal distribution, the two-tailed Wilcoxon matched pairs test or the Mann-Whitney test was used for comparisons between two groups, with the results presented as medians. Statistical significance was defined as a *p*-values less than 0.05 (* *p* < 0.05; ** *p* < 0.01; and *** *p* < 0.001).

## Results

### NcRNAs change the composition of the secretome in a bone remodelling OC/OB co-culture model

**Fig. 1:**
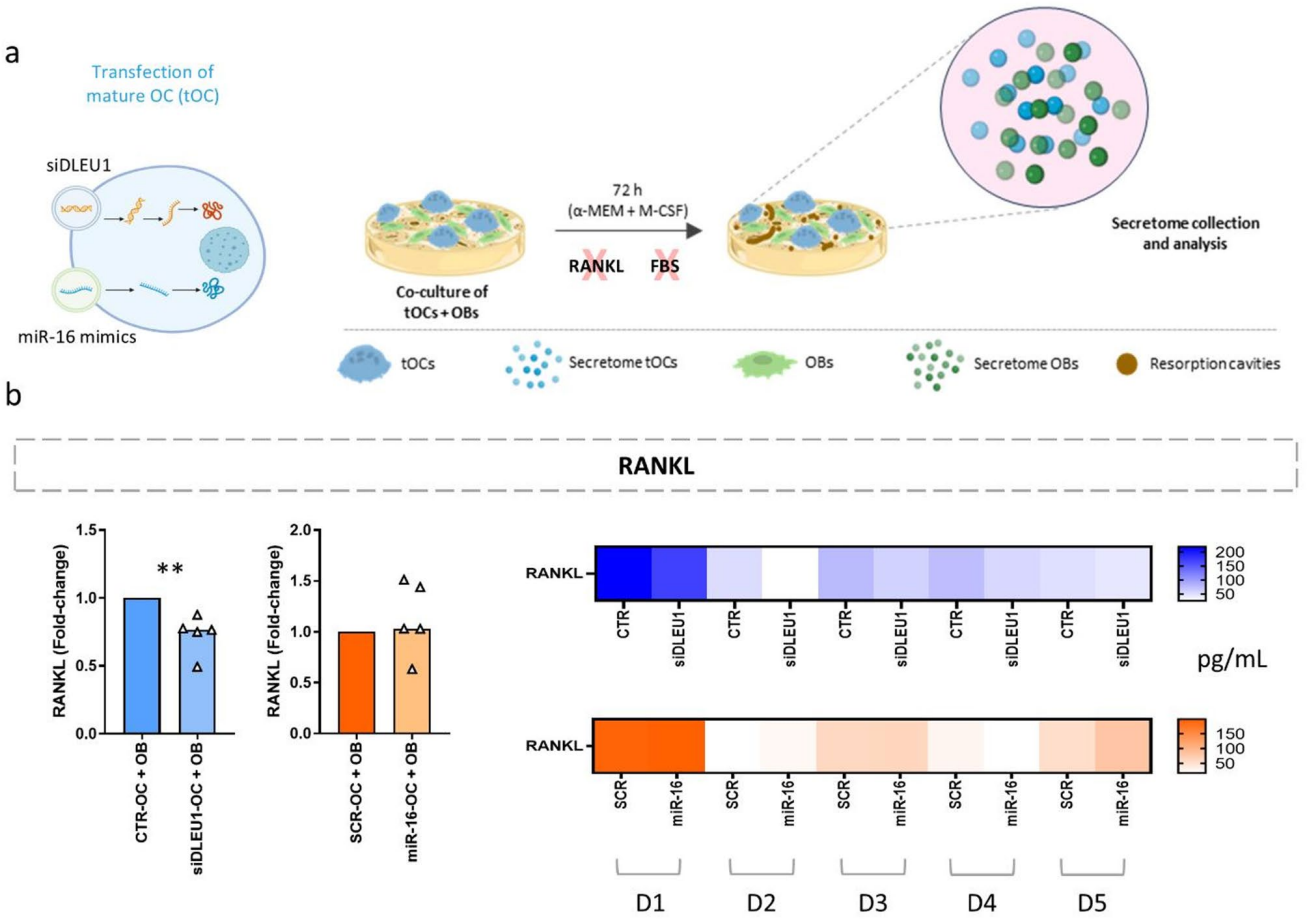
Impact of ncRNA-modulated OCs co-cultured with non-transfected OBs on the levels of RANKL in the secretome. **(a)** Schematic representation of the experimental setup. Osteoclastogenesis was induced, OC were transfected with small interference RNA against *DLEU1* (siDLEU1-OC), or with miRNA-16 mimics (miR-16-OC) or the respective controls, and co-cultured for 3 days on the top of bone slices with primary OBs, without serum and exogenous RANKL. **(b)** Levels of RANKL (*N* = 5) in the secretome of siDLEU1-OC + OB and miR-16-OC + OB co-culture conditions. Each dot represents data normalized to the corresponding control from each OC donor co-cultured with non-transfected OBs. The analysis includes data from five distinct OC donors. Heatmap showing RANKL concentration pattern per donor (D1 through D5). Statistical analysis for panel b was performed using the Mann-Whitney test. The schematic in panel (a) was created using BioRender (https://Biorender.com)

**Fig. 2:**
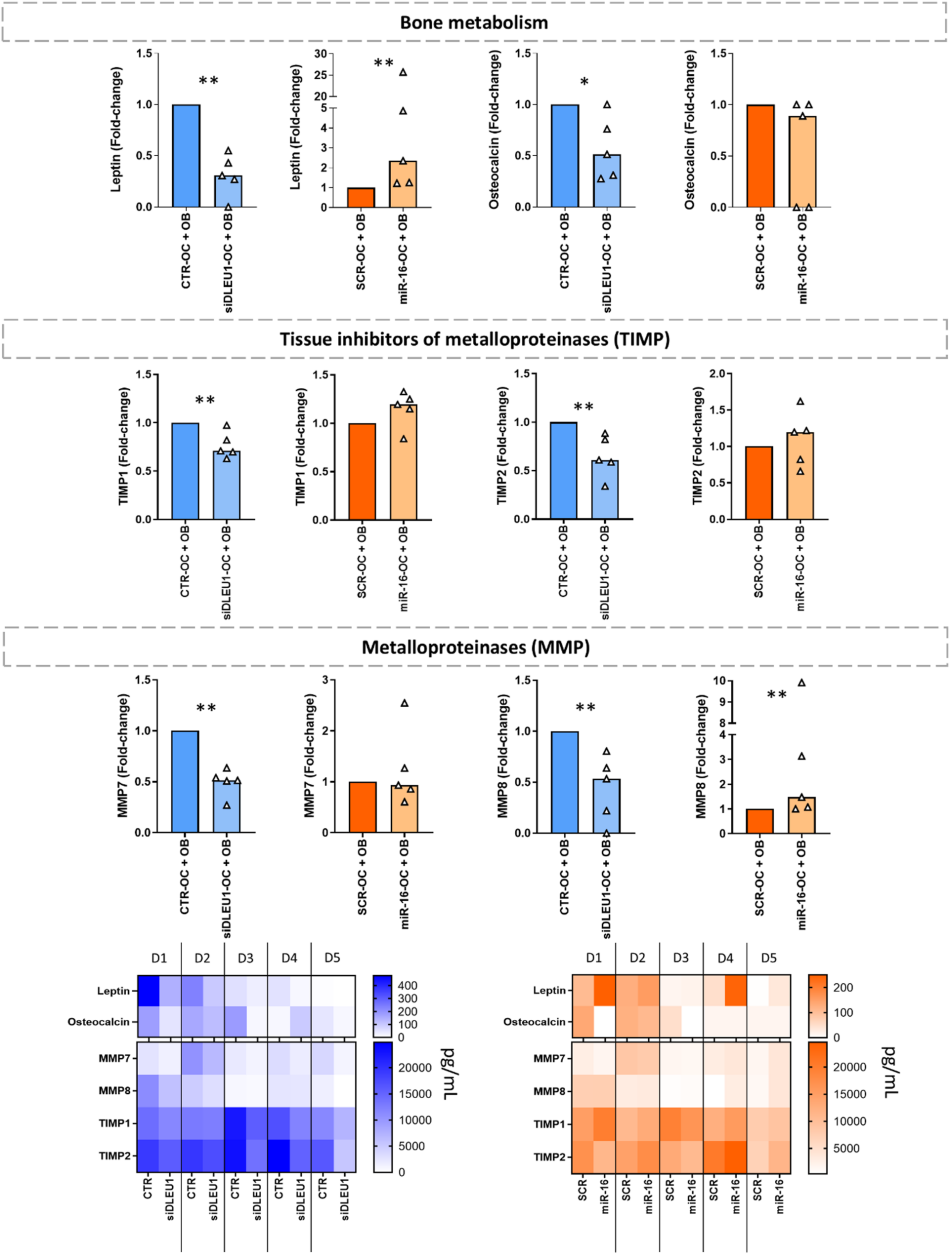
The effect on the secretome of a co-culture model with OBs and ncRNA-modulated OCs. Levels of cytokines/chemokines/proteins in the secretome of siDLEU1-OC + OB and miR-16-OC + OB co-cultures conditions (*N* = 5). Each dot represents data normalized to the corresponding control from eachOC donor co-cultured with non-transfected OBs. The analysis includes data from five distinct OC donors. Heatmaps showing the protein concentration patterns per donor (D1 through D5). Statistical analyses were performed by Mann-Whitney test

For this study, human primary OBs and mature tOCs were co-cultured on cortical bone slices for 72 h in the absence of serum and recombinant RANKL to mimic the bone remodelling process (Fig. 1a). Even without serum and RANKL, both cell types were viable, with OBs being able to proliferate (movie 1), OC being able to resorb bone (movie 2), and with OBs closely interacting with the OCs (movie 3), as expected [31]. Importantly, in addition to potentially influencing the factors secreted by tOCs, modulation of *DLEU1* and miR-16 levels in mature OCs may also affect the behaviour and secretory profile of neighbouring cells. Notably, a significant decrease in RANKL production by OBs was observed in the siDLEU1-OC + OB condition, whereas no significant changes were detected in the miR-16-OC + OB condition (Fig. 1b). To further investigate the impact on the secretome, molecular profiling screen of the tOC/OB secretome was performed (Fig. 2 and Supplementary Figure S2). The results show that the secretome from the co-culture of siDLEU1-OCs with non-transfected OBs contains significantly reduced levels of leptin (*p* < 0.01), osteocalcin (*p* < 0.05), tissue inhibitors of metalloproteinases (TIMP), namely TIMP1 (*p* < 0.01) and TIMP2 (*p* < 0.01), and metalloproteinases (MMPs), namely MMP7 (*p* < 0.01) and MMP8 (*p* < 0.01), compared with the control (CTR-OC + OB) (Fig. 2). On the other hand, for the miR-16-OC + OB condition, the levels of leptin (*p* < 0.01) and MMP8 (*p* < 0.01) (Fig. 2) were increased. The levels of ACTH, FGF-23, insulin, PTH, SOST, TIMP3, TIMP4, MMP12, MMP13 and IL-1β were below the detection range, at the lowest limit of the detection range, or extrapolated in all tested samples; therefore, they were excluded from the analysis. No significant differences were observed in the levels of other proteins, including DKK1, osteopontin, IL-6, OPG, TNF-α, MMP1, MMP2, MMP3, MMP9, and MMP10, across any of the conditions (Supplementary Figure S2). Importantly, although MMPs are expressed by both OCs and OBs, they are a family of enzymes that play a critical role in extracellular matrix (ECM) degradation [50, 51]. Based on these findings, we next investigated whether siDLEU1- and miR-16-primed OCs affected the OB behaviour in the same co-culture model.

### OB migration and ALP activity are affected by siDLEU1-OC

**Fig. 3:**
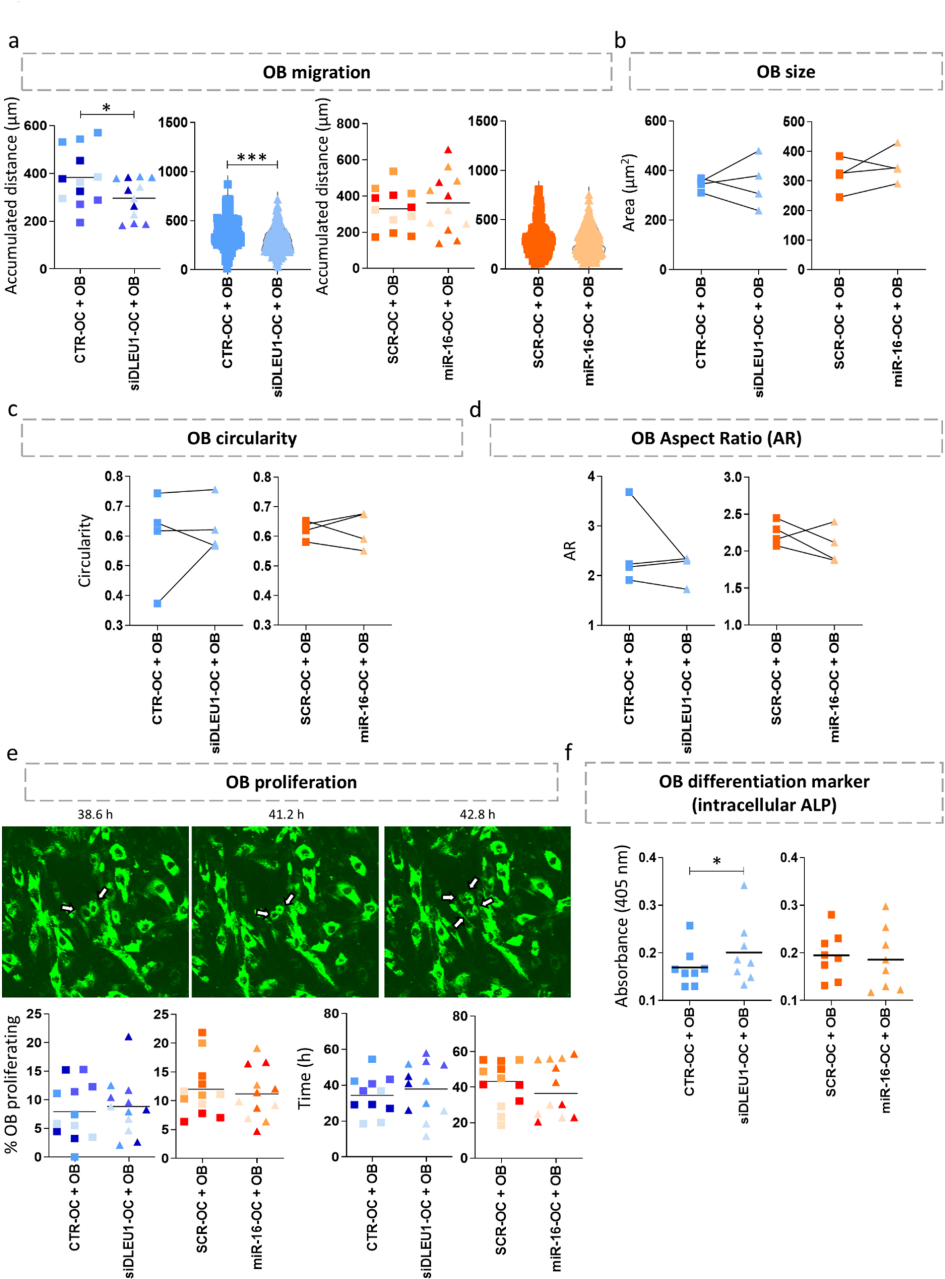
OB behaviour in co-culture with siDLEU1-OCs and miR-16-OCs. **(a)** Accumulated distance travelled by the OBs. Each dot represents the mean distance travelled by the OB per donor (left graphs; *N* = 16). Each dot represents the mean obtained from each video analysed (3 per donor and condition). For each condition, dots with the same shading correspond to videos from the same donor. Distance travelled by individual OBs from a representative donor (right graphs). **(b)** Average size, **(c)** circularity and **(d)** aspect ratio of the OBs (*N* = 4). **(e)** Time-lapse images representative of OB proliferation and quantification of the percentage of OBs proliferating over the course of 72 h, as well as the time of division (*N* = 16). Each dot represents the mean obtained from each video analysed (3 per donor and condition). For each condition, dots with the same shading correspond to videos from the same donor. **(f)** ALP intracellular levels measured in the tOC/OB co-culture system (*N* = 8). Statistical analyses were performed by Student *t* test for panel **a)** mean of accumulated distance, **b)**, **c)**, **d)** miR-16-OC + OB, **e)** % OB proliferating, **e)** time siDLEU1-OC + OB, and **f)**. The Mann-Whitney was used for **a)** distance travelled by individual OBs from a representative donor, and **e)** time miR-16-OC + OB. The Wilcoxon matched-pairs signed rank test was used for **d)** siDLEU1-OC + OB

To test the effect of siDLEU1-OCs and miR-16-OC on OBs, time-lapse recordings were performed for differently fluorescently labelled OBs and tOCs, reflecting a total of 3 456 h (4 independent donors, each with 4 different conditions, and 3 fields recorded per condition). A total of 771, 1 430, 467 and 984 OBs (each of the four independent experiments) were manually tracked to assess the impact on the OBs’ migratory capacity. The results show that OBs co-cultured with siDLEU1-OCs migrated less compared to the control, whereas no differences in migration were observed in OBs co-cultured with miR-16-OCs (Fig. 3a). Regarding the impact on the OB size, circularity, and aspect ratio (AR), no statistical differences were found for both conditions after 72 h in the co-culture model (Fig. 3b, c and d). However, for the miR-16-OC + OB condition, the AR of OBs was decreased in 3 out of the 4 experiments (Fig. 3d and Supplementary Figure S3), suggesting that OBs tended to be less elongated. Next, we tested the potential impact on OBs’ proliferation. When considering the four different conditions (miR-16-OC + OB, siDLEU1-OC + OB, and their respective controls) across 3 fields recorded per condition, a total of 487 cell division events (87, 231, 63 and 106 divisions in each of the independent experiments) were documented in the OBs. After normalizing the number of OBs undergoing division to the total number of OBs visible throughout the 72 h (1 142, 1 859, 656 and 1 408 OBs, respectively), no differences were observed in the percentage of proliferating OBs or the timing of division (Fig. 3e). Considering that ALP is primarily produced by OBs and facilitates the formation of hydroxyapatite crystals, which is an hallmark of bone mineralization [52], we measured the intracellular ALP activity in the tOCs/OBs co-cultures. The results show that ALP activity is significantly increased in OBs co-cultured with siDLEU1-OCs compared with the respective controls, while no differences were found for the miR-16-OCs + OBs co-culture condition (Fig. 3f). Thus, while siDLEU1-OCs reduced the migration of the OBs and enhanced the ALP activity, the migration, size and proliferative capacity of the OBs co-cultured with miR-16-OCs were not affected.

**Silencing** ***DLEU1*** **and overexpressing miR-16 in OC affects OBs affinity for resorbed areas**.

**Fig. 4:**
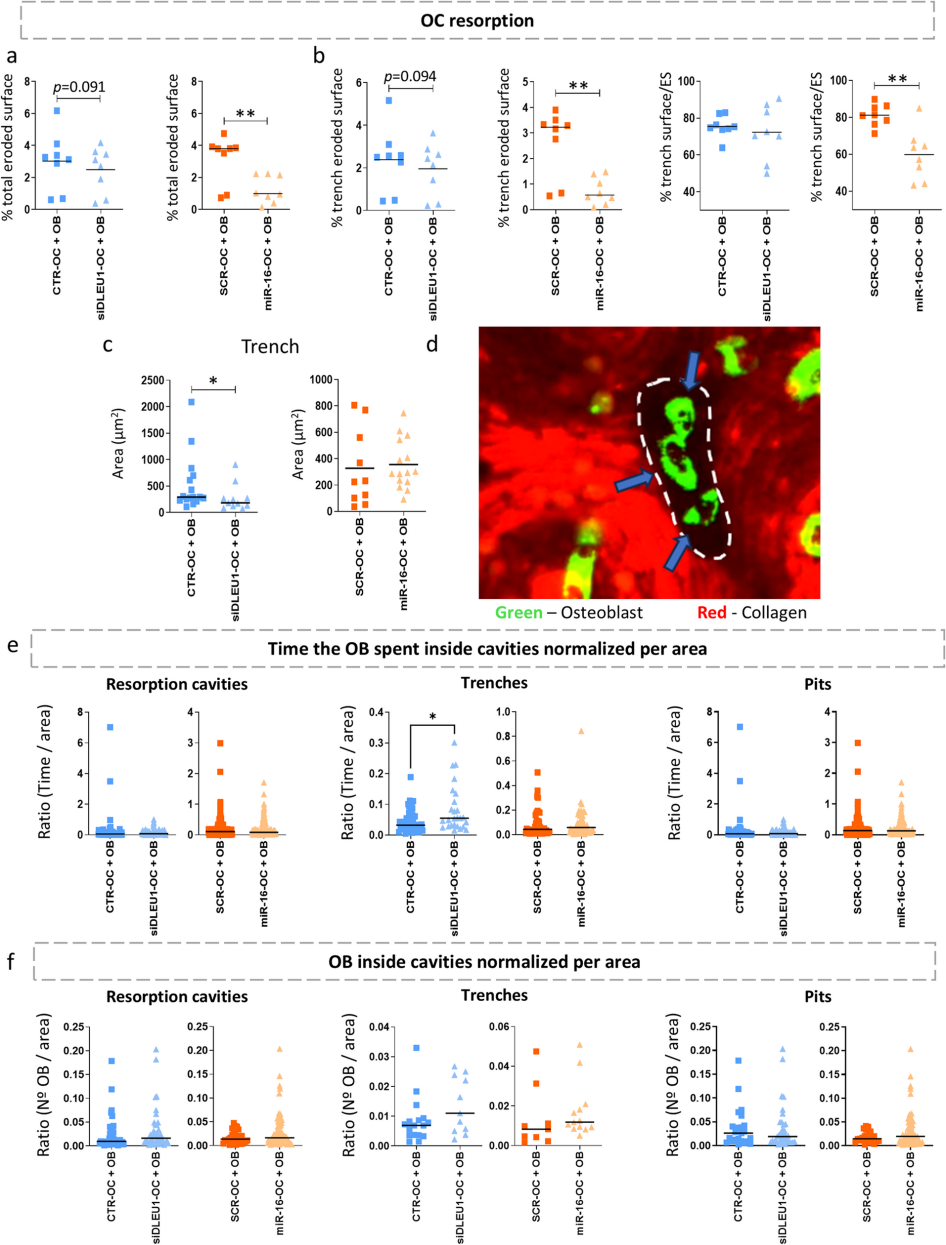
The response of OBs to bone resorption cavities created by OCs is mediated by siDLEU1 and miR-16-mimics. Assessment of the **(a)** total eroded and **(b)** trench surface by OCs after *DLEU1* silencing (siDLEU1) and miR-16 overexpression (miR-16-mimics) (*N* = 8) (5 slices per condition and donor). **(c)** Quantification of individual trench areas from three recordings per condition per donor (data from a representative donor). **(d)** Representative image depicting OBs residing inside resorption cavities. The dashed line delineates a trench-shaped resorption cavity and the arrow indicates OBs (green) actively colonizing the cavity surface (OB - Green and Collagen – Red). **(e)** Time that OBs spent inside the resorption cavities created by the OCs, normalized by area. The ratio between the time spent by each OB inside the cavities and the area of the cavity is represented as a dot. **(f)** Number of OBs that stay inside the resorption cavities performed by the OCs, normalized by area. The ratio between the number of OBs inside the cavities and the area of the same is shown as a dot. Statistical analyses were performed by Student *t* test for panel **(a)** % total eroded surface siDLEU1-OC + OB, **(b)** % trench eroded surface siDLEU1-OC + OB and % trench eroded surface/ES, and **(c)** Area miR-16-OC + OB. The Wilcoxon matched-pairs was used for panel **(a)** % total eroded surface miR-16-OC + OB, and **(b)** % trench eroded surface miR-16-OC + OB. The Mann-Whitney test was used for **(c)** Area siDLEU1-OC + OB, **(e)** and **(f)**

In the co-culture bone biomimetic model, and under deprivation of FBS and exogenous RANKL, the impact of manipulating *DLEU1* and miR-16 levels on resorption activity was assessed across eight different blood donors. Analysis of the percentage of the total eroded surface revealed a trend towards decreased eroded area in the siDLEU1-OC + OB condition, although not statistically significant. Additionally, no specific imbalance was observed between the types of resorption (trenches/pits) (Fig. 4a and b). However, a reduction in the size of the individual trenches was found in the siDLEU1-OC + OB condition (Fig. 4c), while the pit area remained unaffected (Supplementary Figure S4 and S5). This decline aligns with the reduction of MMPs in the secretome (Fig. 2). On the other hand, for the miR-16-OC + OB condition, there is a significant reduction in the percentage of the total eroded surface compared to the control (SCR-OC + OB; Fig. 4a). Also, a shift towards a reduction in the area covered by trenches was detected for miR-16-OCs, in the co-culture system (Fig. 4b). However, no differences were observed regarding the individual area of each trench and pit cavity in the miR-16-OCs + OB condition (Fig. 4c and Supplementary Figure S5). Considering that the bone ECM consists of a complex network of proteins and minerals providing structural support to bone cells, changes in its microarchitecture and composition can influence the behaviour of resident cells, including their recruitment, differentiation and activity. Therefore, we next investigated a potential cell-matrix interaction, focusing on the OBs’ affinity for the resorbed cavities formed by tOCs (Fig. 4d), and whether this response was influenced by the resorption mode. To accomplish this, we normalized both the time spent by each OBs, as well as their number relative to the area of each individual cavity colonized. Time-lapse analysis evidence that the occupancy time of the OBs in pits was higher compared to trenches (Fig. 4e). Specifically, results show that the OBs spent more time within the trenches formed by the siDLEU1-OC (Fig. 4e), suggesting that either the microarchitecture or the matrix composition may influence this response. In contrast, no differences were found for the time spent by OBs within miR-16-OC-derived cavities, irrespective of the resorption mode (Fig. 4e). Meanwhile, the number of OBs colonizing the resorption cavities indicates a greater frequency of colonization of pits compared to trenches (Fig. 4f). No differences in the number of OBs inside the cavities for either the siDLEU1-OC + OB or miR-16-OC + OB conditions were detected, compared with their respective controls (Fig. 4f).

## Discussion

While significant progress has been made in osteoporosis treatment through monoclonal antibodies and hormone therapies, the current therapeutic strategies remain predominantly focused on targeting OCs, rather than OBs, given their central role in the onset of osteoporosis. Surprisingly, the number of studies addressing the role of ncRNAs in OC function is limited, particularly those using human primary OCs. This is especially critical for lncRNAs, which, compared with mRNA, exhibit low sequence conservation across species [53, 54], underscoring the importance of human-based studies for translational relevance. Due to their limited lifespan and the technical challenges associated with genetic manipulation, primary human OCs are often replaced by the murine RAW 264.7 cell line in ncRNA research. While this model offers practical advantages, it also presents significant limitations, with the RAW 264.7 cells exhibiting distinct transcriptional profiles [55] and non-specific differentiation potential into multiple multinucleated cell types beyond osteoclasts [56]. Therefore, the use of primary human OCs is a major strength of our study.

Previous work from our group showed that *DLEU1* expression is significantly upregulated during OC differentiation, consistent with a pro-osteoclastogenic role. Conversely, miR-16 expression was downregulated, supporting its function as a negative regulator of osteoclastogenesis. Moreover, we demonstrated that the silencing of *DLEU1* and the overexpression of miR-16 affected the fusion capacity of preOCs and the resorptive capacity in multinucleated mature OCs in mono-culture [18]. In line with these results, the levels of miR-16 were decreased in the serum of patients with fractures [57] and bone tissues from osteoporotic patients and OVX mice [58]. Building on these findings, and to investigate the impact of siDLEU1- and miR-16-engineered OCs on bone remodelling, here we propose to use a co-culture model of OCs and OBs seeded onto bone slices, proven to mimic the interaction between these cells in vivo [2, 59, 60]. Unlike 2D mono-cultures, which lack 3D microenvironmental cues necessary to replicate the complexity of human tissues and organs, more sophisticated and physiologically relevant models are being adopted. For this reason, and in an attempt to use an in vitro bone biomimetic system, in this manuscript, we co-cultured human primary OCs (engineered either with siDLEU1 or miR-16 mimics) with OBs and seeded them onto bone slices to investigate the responses of OBs to ncRNA-engineered-OCs. Interestingly, compared to the mono-culture system used in our previous work [18], a significant reduction on the OCs resorptive activity was observed, as evidenced by a reduction in the total area resorbed in the miR-16-OCs + OB condition. These findings strengthen the importance of using more elaborate models to better understand the possible influence of the tissue microenvironment.

Moreover, the absence of recombinant RANKL in the culture medium implies that the RANKL required by the OCs is naturally produced by the OBs, thereby reflecting a more physiological context. The absence of serum and exogenous RANKL was a deliberate step to isolate specific cellular interactions and to study the direct OB-OC coupling mechanisms. This approach allows to eliminate confounding serum-derived factors that could mask cell-autonomous communication and force dependence on physiological OB-derived RANKL to sustain OC activity. Still, while this represents a valuable in vitro model for dissecting OB-OC interactions, it may underestimate coupling magnitude compared to in vivo settings, where the presence of other cell types and molecules in the bone microenvironment also interferes with the OC/OB dynamic communication. In our results, RANKL production, essential for OC activity, is impaired in the secretome from the siDLEU1-OC + OBs condition. Importantly, the decrease in RANKL expression is associated with an increased osteogenic potential, which is in line with the increased ALP activity observed for this condition. Furthermore, this co-culture was entirely performed in the absence of serum, as its addition, along with recombinant RANKL, has been reported to hinder any stimulation of OC bone resorption by the OBs [31]. Recently, using the same co-culture model, Panwar et al. reported an enhanced OB recruiting, differentiation, and activity in short and incomplete trenches during partial cathepsin K inhibition [41]. Consistently, our study demonstrates increased ALP activity in the siDLEU1-OC + OB condition, where the size of each individual trench was smaller.

Furthermore, we observed OBs in direct contact with OCs, as well as trailing behind actively resorbing OCs, consistent with findings reported in other studies [2, 61]. This coupling may result from the secretion of clastokines by OCs [62–64], which affects the neighbouring cells, or by the attraction of OBs to the newly exposed collagen I [31, 65, 66]. We found that modulation of *DLEU1* and miR-16 levels in mature OCs alters the secretome composition in the OC/OB co-culture model. Although ncRNA modulation specifically targeted OCs, the resulting changes in the secretome composition may not be solely attributed to proteins differentially secreted by the OCs, but also to OB responses to these alterations, as well as to proteins/factors released from the remodeled matrix. Moreover, in our co-culture model, proteins are being secreted and consumed simultaneously, which further reflects the complex interactions occurring during the OC-OB cross-communication. For instance, in our previous study, proteomic analysis revealed that SPP1 (osteopontin) was upregulated in siDLEU1-OCs compared to CTR-OCs, in mono-cultures [18]. However, this upregulation was not maintained in co-culture with OBs, suggesting that OBs may modulate or buffer the molecular effects of OC-specific ncRNA modulation within a more complex bone-like environment. This finding highlights the importance of context-dependent regulation and underscores the need to interpret mono-culture data with caution when extrapolating to multicellular systems.

Regarding MMPs, the reduction of their levels in the secretome for the siDLEU1-OC + OB condition might support the formation of smaller individual trenches by siDLEU1-OC, as this family of proteinases cleaves ECM proteins [59] and MMP inhibition results in decreased collagen solubilization in the resorption zone of explanted bone [67]. Interestingly, while studies using bone explants demonstrate that MMPs are involved in the bone resorption process [60, 67, 68], the inhibition of MMPs in OC mono-cultures [31, 69] and in OCs cultured on dentine slices [70] did not affect their resorptive capacity, suggesting that the use of MMP inhibitors does not impact the OCs resorptive activity in the absence of other bone cells. Besides the OCs, cells from the OB lineage have been described to express MMPs, in particular MMP13 [59]. Pirapaharan et al. reported that the presence of OBs favoured bone resorption, especially by augmenting the formation of trenches, resulting in larger trenches and increasing the number of resorption events regardless of the resorption mode [31]. Remarkably, MMP8 has been reported to enhance the mobilization of human hematopoietic stem cells [71]. Although prevenient from other lineage, in this study, we also observed an impairment of the migratory ability of the OB co-cultured together with siDLEU1-OCs, which aligns with the reduced levels of MMP8 in the secretome for this condition. Therefore, besides matrix solubilisation, MMPs should be considered in a broader context, including their activity during OC recruitment [72–74], MSC recruitment [75], OB survival [76], coupling between bone formation and destruction [31], and destruction/bioactivation of cytokines and/or growth factors. Moreover, osteocalcin levels are decreased in the siDLEU1-OC + OB condition, what might suggest a negative impact on the OBs co-cultured with the tOCs. Nevertheless, although early studies suggest that circulating osteocalcin originates from bone-forming cells, recent works on metabolic bone diseases evidence that osteocalcin can also come from bone breakdown and it may be released from the bone matrix during osteoclastic bone resorption [77]. In fact, the amount of osteocalcin released into the medium increases in the presence of resorption stimulators, in contrast to lower levels detected with resorption inhibitors. Its concentration in the medium is also positively correlated with C-terminal telopeptide of type I collagen (CTX), a marker of bone resorption [77]. Therefore, osteocalcin can also be used as a marker of bone resorption in vitro and its circulating levels should be considered as a marker of bone turnover rather than bone formation. In our model, given that osteocalcin is highly conserved among vertebrates and is known to be released during bone resorption, the reduced osteocalcin levels detected in the secretome of siDLEU1-OC + OB cultures, together with the observed increase in ALP levels and decreased RANKL production by the OBs, may suggest impaired OC activity and reduced matrix degradation, rather than a result of OBs activity. Besides a possible paracrine effect of the engineered OCs on the OBs, the composition and microarchitecture of the bone matrix are also crucial for OB function, influencing their adhesion, migration, differentiation, and overall activity by providing structural support and biochemical signals [41, 78–82]. Proteins such as collagen type I and osteopontin have been shown to promote OB adhesion, proliferation and differentiation, all of which are essential for effective bone formation and mineralization [79]. Other ECM components involved in OB-driven bone formation include osteonectin and bone sialoprotein [79]. The synergy between matrix components ensures that OBs can efficiently build new bone tissue. Moreover, in the context of bone regeneration, the surface topography (e.g., roughness and stiffness), in addition to substrate composition, has been shown to influence the OB-mediated bone production [80, 83]. Moreover, the type of resorption cavity, particularly their different biochemical and topographical landscape, has also been described to play distinct roles in OBs behaviour, namely OB engagement and differentiation [41], underscoring the importance of evaluating pits and trenches separately in bone remodelling studies. While OCs making trenches are typically associated with high cathepsin K activity, resulting in longer, deeper cavities devoid of residual demineralized collagen and indicative of complete matrix degradation, pits, in contrast, show an accumulation of demineralized collagen [24, 84, 85]. Interestingly, a recent study by Panwar et al., demonstrated that both the average occupancy and individual time of OBs in pits were higher than in trenches, evidencing that the eroded matrix of pits is more attractive to OBs than that of trenches [41]. This finding underscores the importance of distinguishing between pit and trench resorption, particularly in pathophysiological conditions where bone resorption is elevated and trench formation predominates [19, 45]. In the present study, we observed that the engagement of the OBs with the resorption cavities was also affected in the siDLEU1-OCs + OB condition, specifically with the trenches, suggesting that this interaction might also be regulated by ncRNAs. It is important to point out that alterations in the area of each individual cavity colonized was taken into consideration to remove the potential influence of the cavity size on the results. These results support the hypothesis that either the microarchitecture or the composition of the newly exposed matrix is influencing the OBs response. Notably, the trenches formed by *siDLEU1*-OCs were smaller compared to controls, which can indicate incomplete collagen degradation and presence of residual collagen fibers. This might explain alterations in the engagement of the OBs since collagen fibers exert strong haptotaxis on OBs compared to other bone matrix proteins in vitro [66] and might explain the larger OB occupancy of pits compared to trenches [41]. Future studies exploring the matrix composition of trenches generated by siDLEU1-OC should be performed to confirm our hypothesis and understand the cues that generate a “permissive” matrix for OB activity.

Based on our findings, suppression of *DLEU1* and/or overexpression of miR-16 reduces bone resorption without compromising bone formation, an advantage over conventional anti-resorptive agents like bisphosphonates. However, clinical translation faces significant challenges, which include the development of reliable delivery systems capable of tissue-specific targeting to minimize systemic effects, ensuring the specificity to avoid potential off-target effects and immune response, and stability of RNA-based therapeutics in vivo to ensure protection from degradation. For bone regeneration, a potential strategy for siRNA or miRNA-mimics delivery would be the use of gene-activated matrices (GAMs) [86, 87], which could be engineered to release nucleic acids in a controlled fashion, thereby modulating OC/OB behaviour. Alternatively, injectable hydrogels with oligonucleotides encapsulated have also emerged as a promising approach to treat bone defects [88]. Given the multifactorial nature of osteoporosis, the combination of therapies targeting both OCs and OBs may offer enhanced efficacy in managing the disease, resulting in better outcomes in terms of fracture prevention and/or treatment.

### Supplemental material and methods

#### Transfection of OCs with SiGLO

PreOCs (on day 3) and matured OCs (on day 8) were subjected to transfection with CTR lncRNA (CTR; 25 nM) and siGLO (25 nM; Dharmacon – Horizon Discovery). The culture media with the transfection reagent was changed after 6 h and cells were fixed with 40 g/L PFA at day 5 (for the OC transfected at day 3) and day 12 (for the OC transfected at day 8). Samples were permeabilized with 0.1% (v/v) Triton-X100, blocked with 50 g/L bovine serum albumin (BSA), and then incubated for 20 min with Alexa Fluor™ 488 Phalloidin (1:200, A12379, ThermoFisher Scientific) and 5 min with DAPI (1:1 000, 62248, ThermoFisher Scientific). The examination of fluorescence emitted at 547 nm (red) from siGLO inside the cell was assessed using fluorescence microscopy (ZOE Fluorescent Cell and Leica DMI6000 FFW). Image analysis was performed using Image J 2.0 software. Cell viability was evaluated through calcein/Propidium Iodide (PI) staining. Briefly, cells were treated with 2 µg/mL calcein and incubated for 20 min in a controlled environment at 37 °C with 5% (v/v) CO_2_. Subsequently, a 1 µg/mL PI solution was added for an additional 10 min. Following this, the cells underwent a thorough wash and images were captured using the ZOE Fluorescent Cell imaging system.

### Supplemental results

#### OCs were efficiently transfected with SiGLO

OCs and their precursors (preOC) are resistant to transfection, which presents a unique set of additional challenges associated with their manipulation. Transfection of these cells commonly results in high cell death and unsatisfactory transfection efficiencies. This challenge is even more pronounced when considering mature OCs. To facilitate the monitoring of transfection efficiency, we transfected preOCs (day 3) and matured OCs (day 8) with siGLO oligonucleotides. The delivery and retention of siGLO was monitored using fluorescence microscopy for preOCs immediately after removal of the transfection mix/particle loading (data not shown), on day 5 (Supplemental Fig. S1a) and day 9 of the differentiation process (data not shown). For mature OCs, the assessment was conducted right after removal of the transfection mix (data not shown) and on day 12, under M-CSF and RANKL supplementation (Supplemental Fig. S1b). The transfection efficiency was evaluated at day 5, for preOC (48 h post-transfection) and day 12, for mature OC (72 h post-transfection), by counting intracellular siGLO-positive cells (Supplemental Fig. S1c; left). Results show that siGLO was successfully delivered to both pre and matured OCs for the two donors tested (Supplemental Fig. S1c; right). Simultaneously, no cell differences regarding cell death were observed at day 12 for mature OCs transfected at day 8 (Supplemental Fig. S1d).

## Electronic supplementary material

Below is the link to the electronic supplementary material.

Supplementary Material 1

Supplementary Material 2

Supplementary Material 3

Supplementary Material 4

Supplementary Material 5

Supplementary Material 6

Supplementary Material 7

Supplementary Material 8

Supplementary Material 9

Supplementary Material 10

Supplementary Material 11

Supplementary Material 12

## Data Availability

No datasets were generated or analysed during the current study.
