# Supplementary figures and images for: Non-coding RNA modulation in osteoclasts and its implications for osteoblast lineage cell behavior in a co-culture system
Source: Cell Commun Signal. 2025 Aug 12;23:370. doi: 10.1186/s12964-025-02324-7 (PMC12341292; doi:10.1186/s12964-025-02324-7)

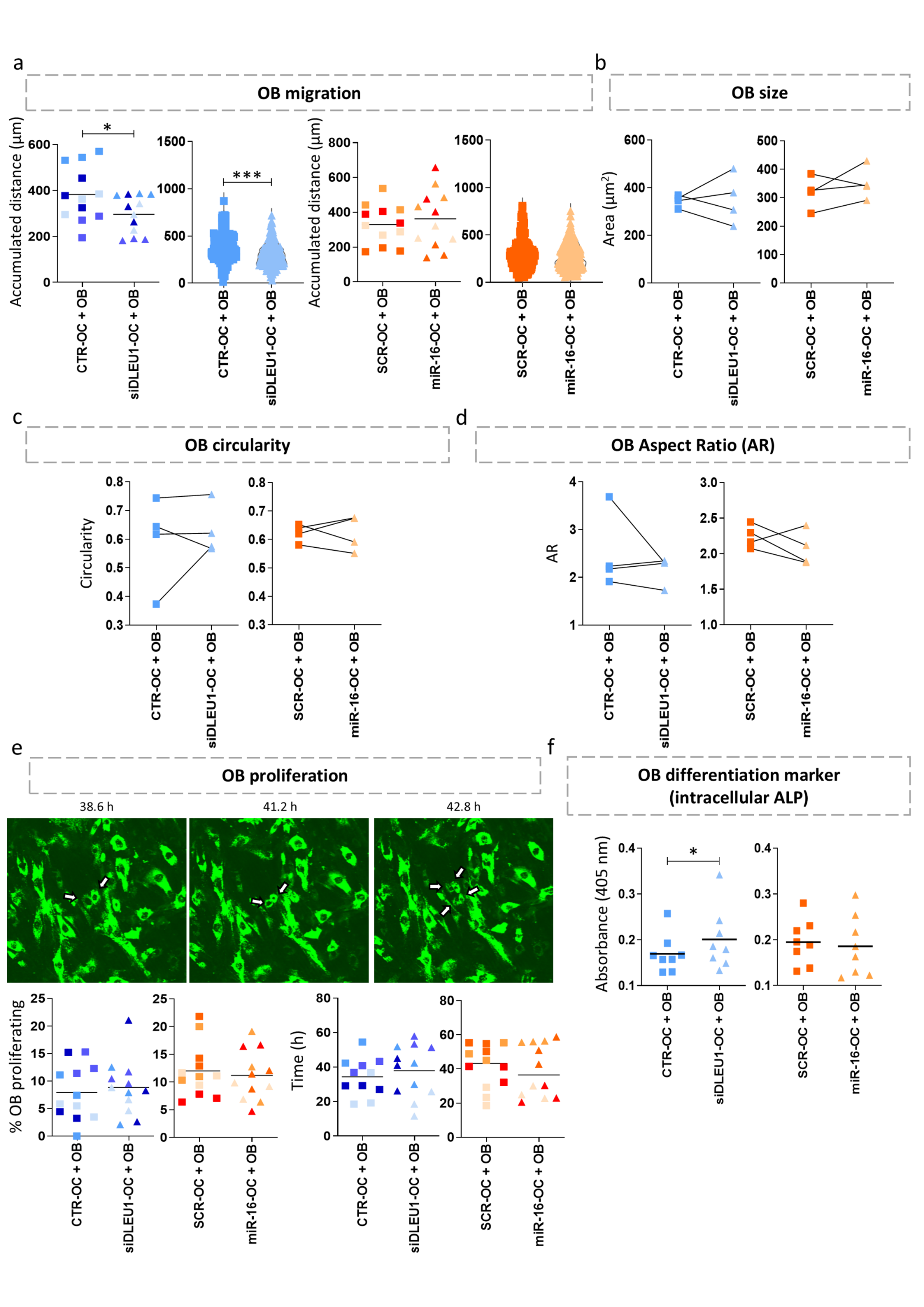

Supplement: Supplementary file 4 — Supplementary Material 4 [file 12964_2025_2324_MOESM4_ESM.tif]

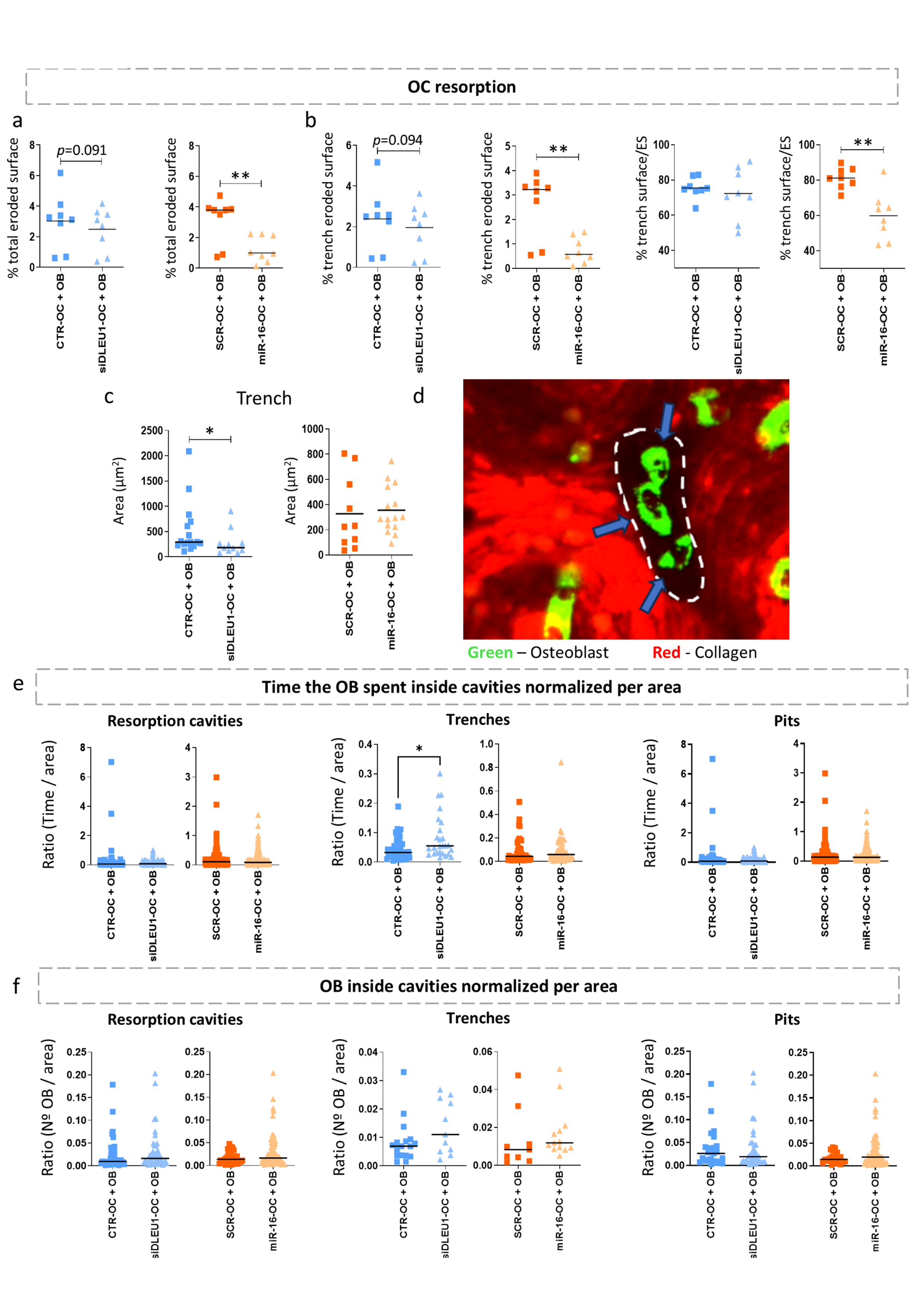

Supplement: Supplementary file 5 — Supplementary Material 5 [file 12964_2025_2324_MOESM5_ESM.tif]

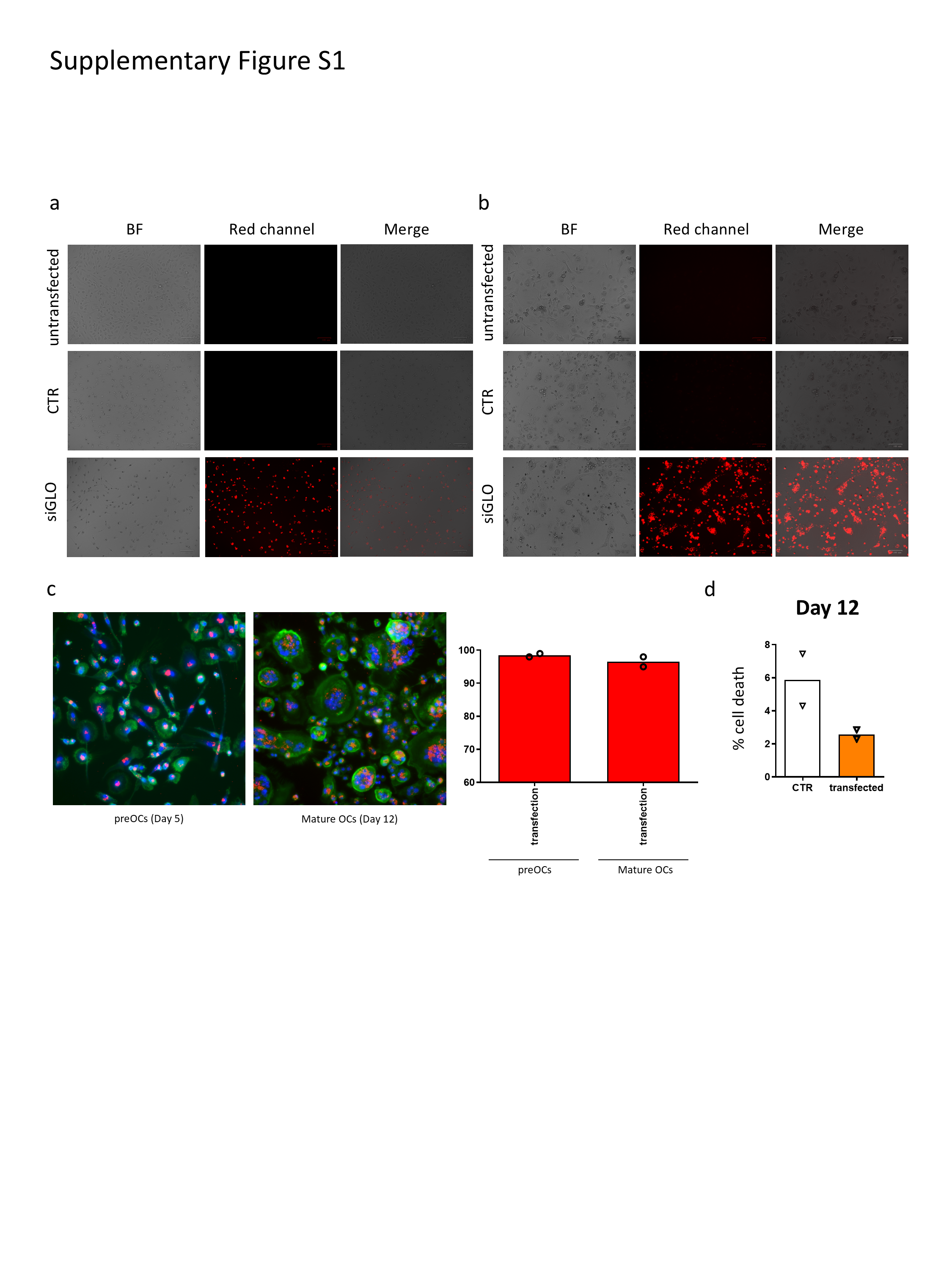

Supplement: Supplementary file 6 — Supplementary Material 6 [file 12964_2025_2324_MOESM6_ESM.tif]

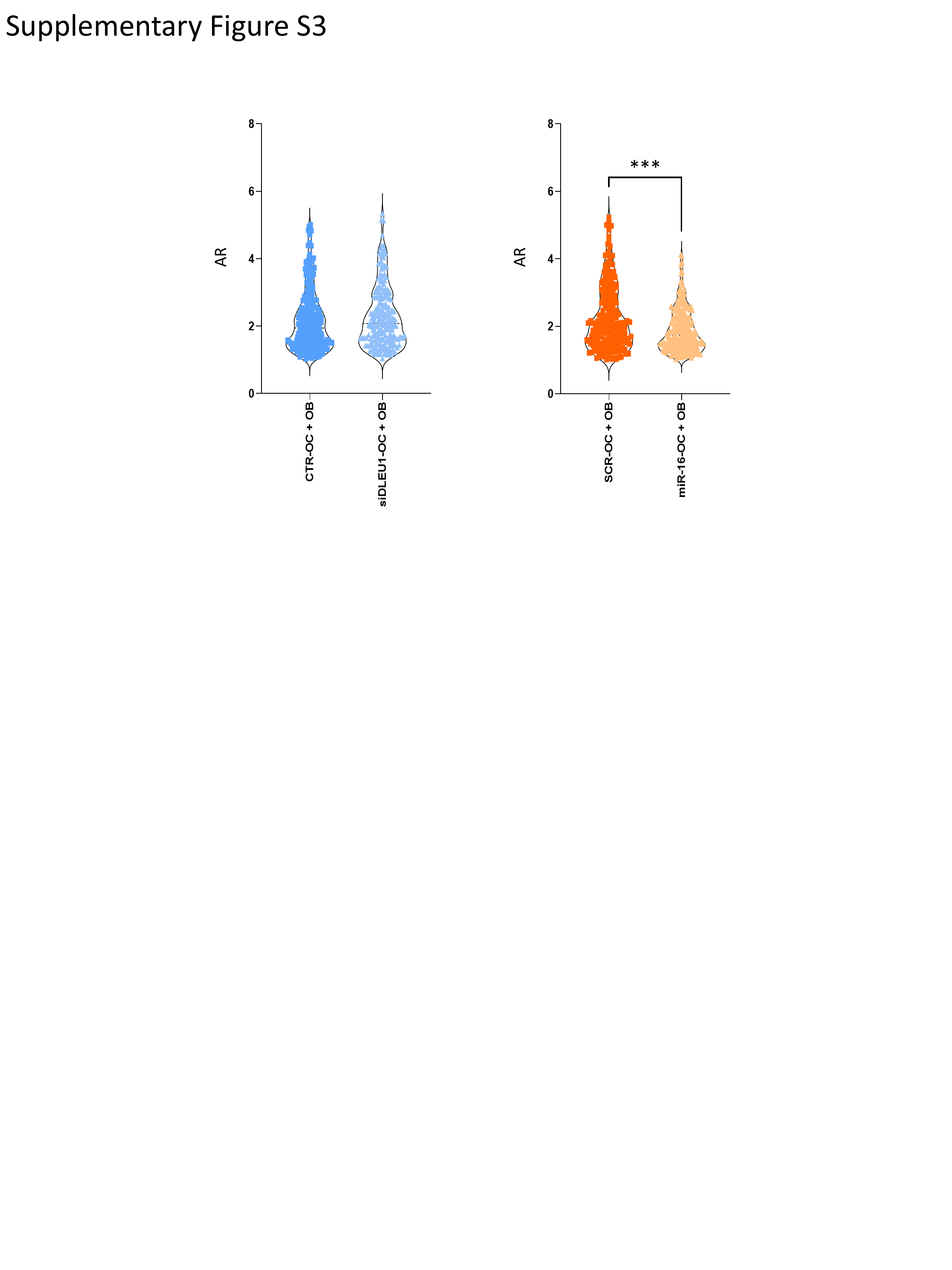

Supplement: Supplementary file 7 — Supplementary Material 7 [file 12964_2025_2324_MOESM7_ESM.tif]

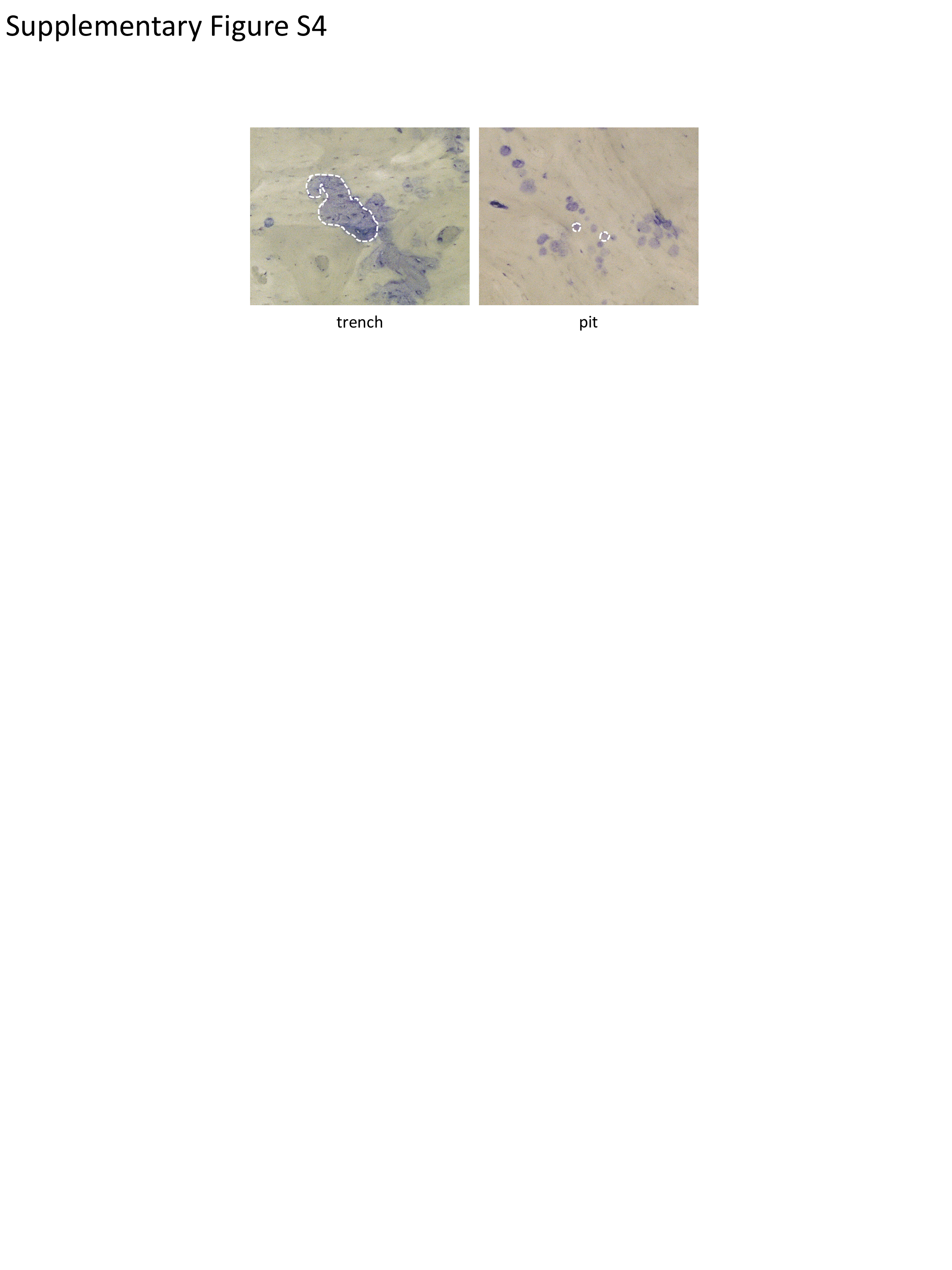

Supplement: Supplementary file 8 — Supplementary Material 8 [file 12964_2025_2324_MOESM8_ESM.tif]

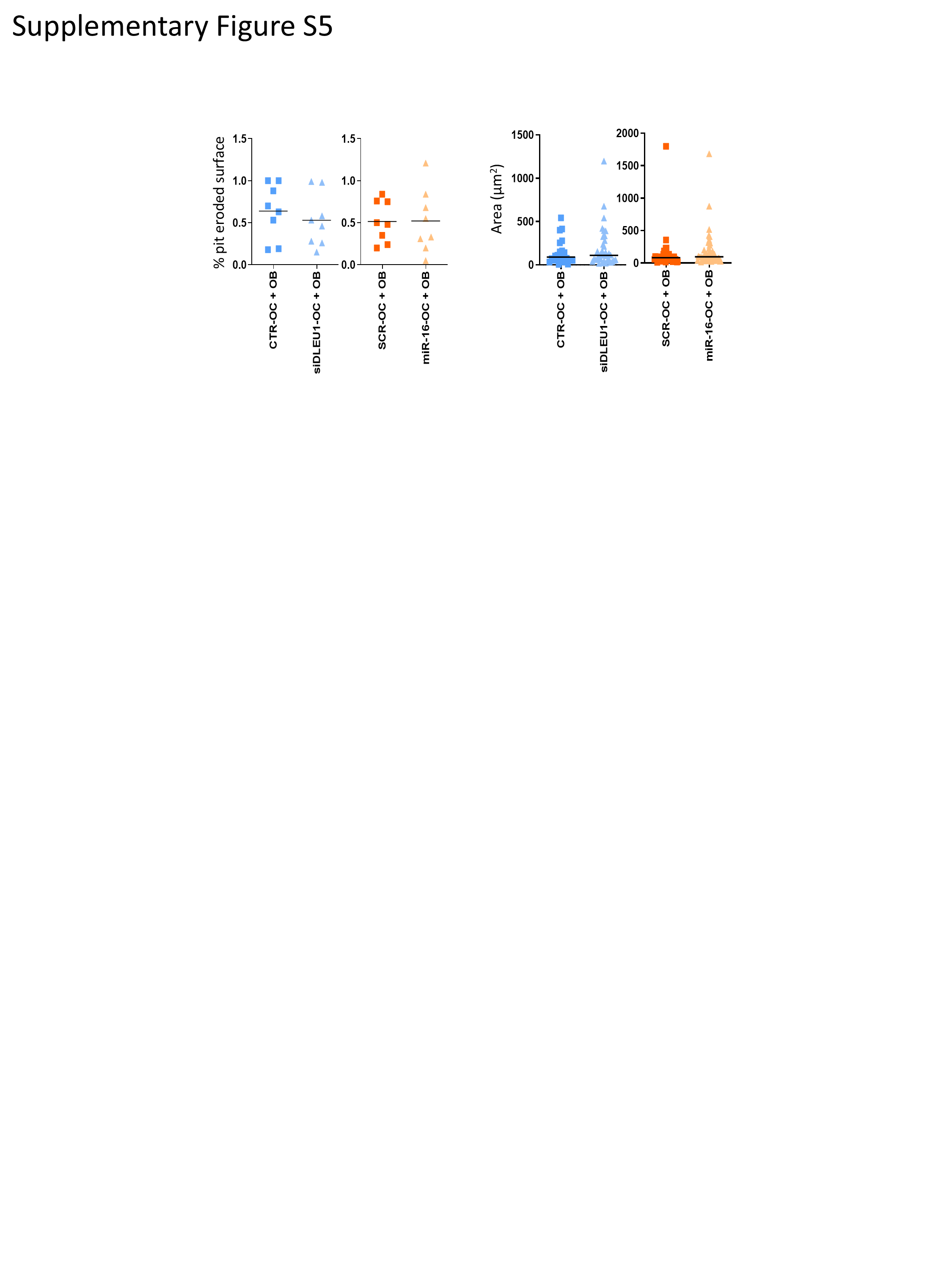

Supplement: Supplementary file 9 — Supplementary Material 9 [file 12964_2025_2324_MOESM9_ESM.tif]

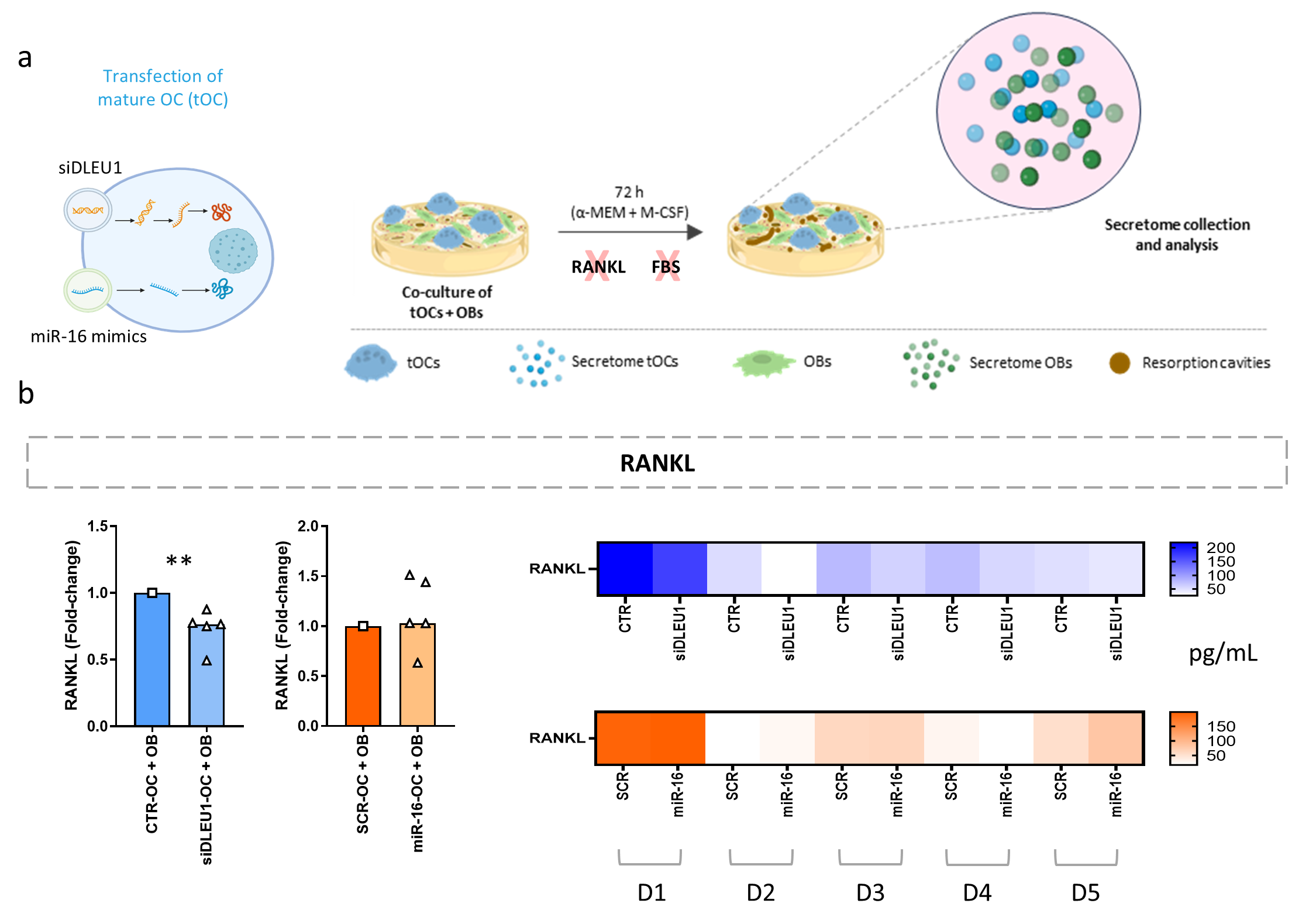

Supplement: Supplementary file 10 — Supplementary Material 10 [file 12964_2025_2324_MOESM10_ESM.tif]

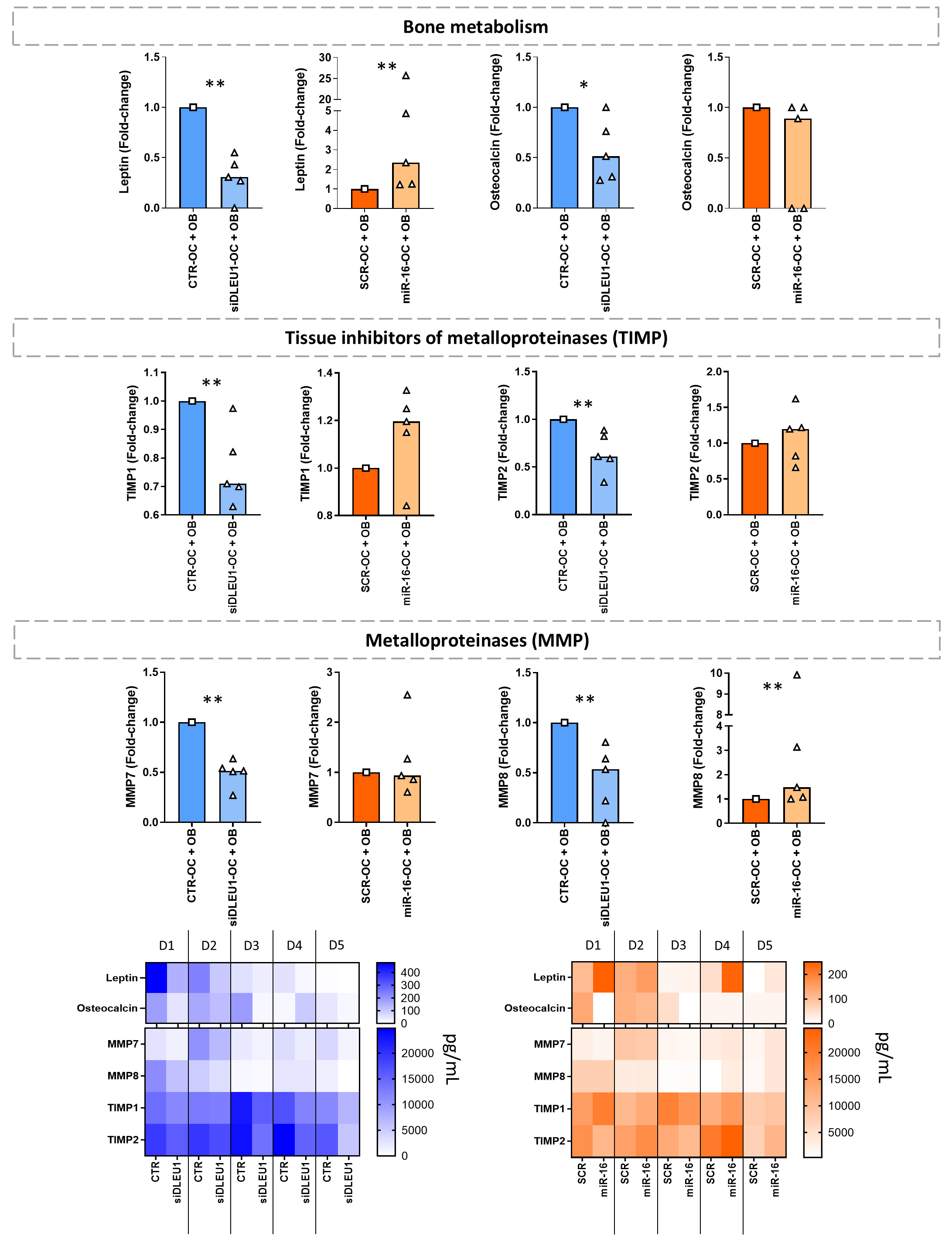

Supplement: Supplementary file 11 — Supplementary Material 11 [file 12964_2025_2324_MOESM11_ESM.tif]

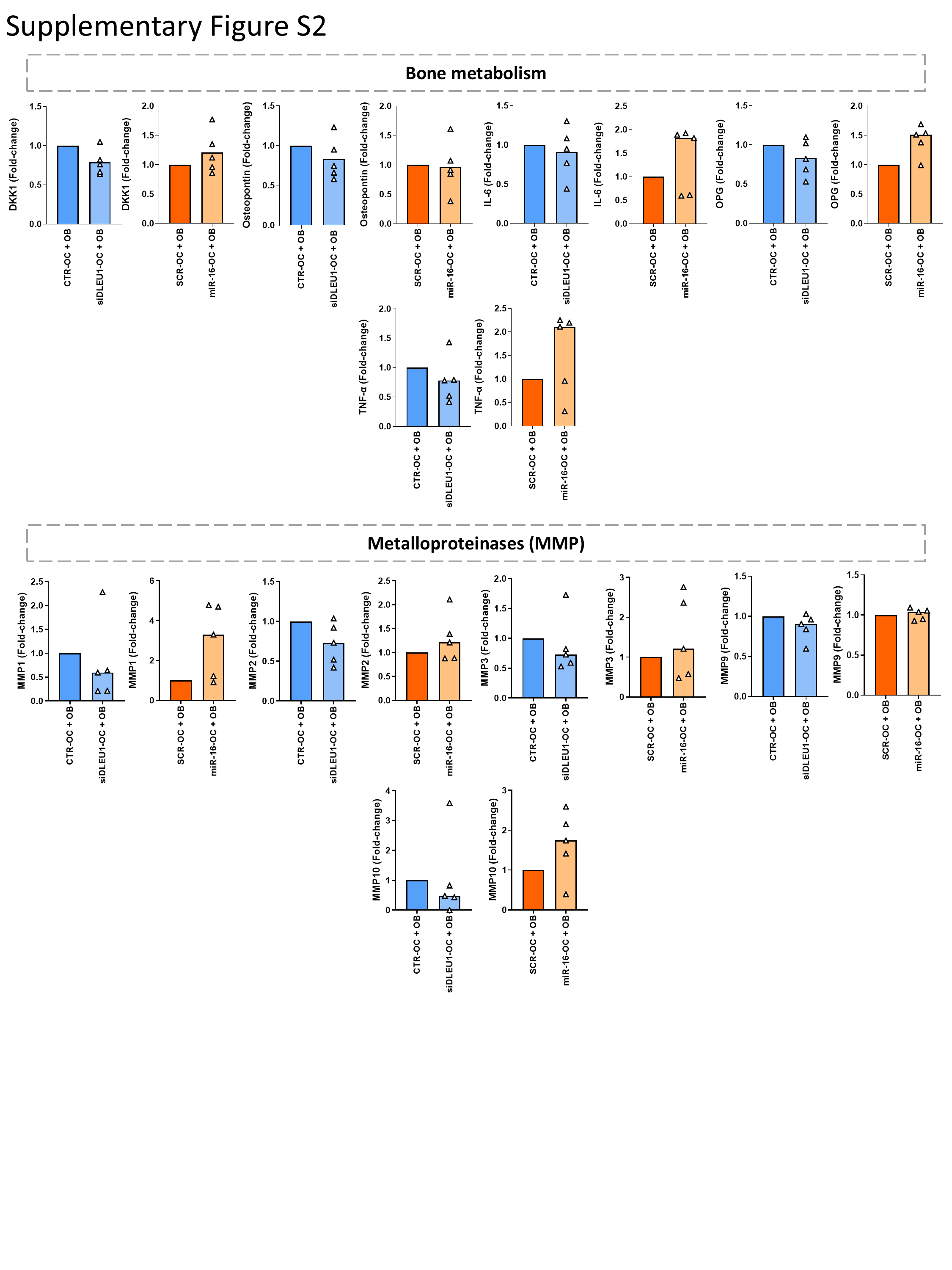

Supplement: Supplementary file 12 — Supplementary Material 12 [file 12964_2025_2324_MOESM12_ESM.tif]
